# Draft genomes of five bacteria isolated from *Carcharodon carcharias* (white shark) and *Carcharhinus brachyurus* (bronze whaler shark)

**DOI:** 10.1128/mra.00226-25

**Published:** 2025-06-30

**Authors:** Emma N. Kerr, Ryan D. Hesse, Jessica A. P. Carlson-Jones, Bhavya Nalagampalli Papudeshi, Paul A. Butcher, Michael P. Doane, Elizabeth A. Dinsdale

**Affiliations:** 1 Flinders Accelerator for Microbiome Exploration, Flinders University 1065, Adelaide, South Australia, Australia; 2 New South Wales Department of Primary Industries and Regional Development, National Marine Science Centre 549395 , Coffs Harbour, New South Wales, Australia; University of Southern California, Los Angeles, California, USA

**Keywords:** Elasmobranch, shark, microbiome

## Abstract

Here, we isolated five bacterial species from the skin and cloaca of *Carcharodon carcharias* (white shark) and *Carcharhinus brachyurus* (bronze whaler or copper shark).

## ANNOUNCEMENT

Shark microbiology originated in response to shark bites (1). However, microbes are integral in shark health and ecology (2, 3). Elasmobranchs support biodiversity in marine ecosystems but are globally threated by overfishing and biomagnification of heavy metals (4–6). Biomagnification is reflected in shark microbial communities where heavy metal resistance genes are enriched (7–10). We sequenced the genomes of bacterial isolates from *Carcharodon carcharias* (white shark) and *Carcharhinus brachyurus* (bronze whaler shark).

Sharks were caught on SMART drumlines at Port Macquarie, New South Wales (NSW DPI scientific [Ref. P01/0059(A)], Marine Parks [Ref. P16/0145-1.1], and Animal Care and Ethics [ACEC Ref. 07/08]) (11, 12). Swabs were collected from the skin and cloaca, placed in a sterile seawater-filled microfuge tube and transported on ice. The bacterial slurry (50 µL) was plated on Thiosulfate–citrate–bile salts–sucrose agar (Bacto Laboratories, Australia) and incubated at 18°C until colonies formed. Colonies were isolated and picked for DNA extraction using the Nucleospin Tissue Kit (Macherey-Nagel, Germany). DNA concentrations were quantified with a Qubit 4 fluorometer (Invitrogen, USA). Libraries were prepared with raw DNA without size selection using the Nanopore Rapid Barcoding Kit and sequenced on the MinION (flow cell R9.4.1) (Oxford Nanopore, United Kingdom). Default parameters were used for all bioinformatics tools. Guppy basecalling (V 6.5.7) converted raw signal to base pairs (Oxford Nanopore Technology). Hybracter (V 0.10.1) quality-controlled sequences and assembled contigs (13). Average coverage is the mean coverage of all contigs in a genome. Taxonomic classification was determined using a BLASTN search of the entire genome sequence (14) and Bakta (V 1.10) for functional annotation (15). Bandage (v0.8.1) was used to visualize genomes (16).

**Fig 1:**
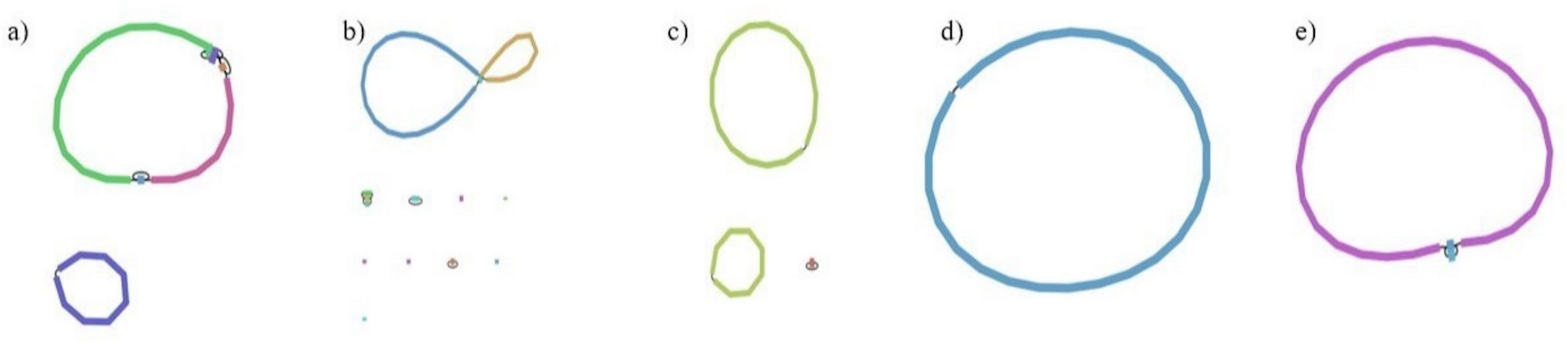
Visualization of genome assemblies. (a) *Vibrio toranzoniae* strain ENK1. (b) *Aliivibrio fischeri ES114* strain ENK5. (c) *Vibrio atlanticus* strain ENK4. (d) *Shewanella* sp. *WPAGA9* strain ENK2. (e) *Shewanella woodyi* strain ENK6.

Genomes were complete and contained 4,750,424 to 5,949,190 base pairs in 1–11 contigs (Fig. 1, Table 1). Antimicrobial and heavy metal resistance genes were present in all genomes. Cloaca isolates have class A beta-lactamase, heavy metal efflux pump, trimethylamine-N-oxide (TMAO) reductase, and urease genes which are lacking from skin isolates. TMAO and urea are used to maintain osmotic balance by Elasmobranchs (17). Presence of these genes may indicate compounds utilized by shark microbes. Cation Diffusion Facilitator (CDF) family efflux transporter DmeF gene was present in skin isolates and may provide resistance to heavy metals (18).

**TABLE 1:** Isolate genome statistics

Species	Host	Genome length (bp)	Number of reads	Sequence N50	Contigs	Assembly N50	GC content (%)	Average coverage	Annotated genes	SRA	GenBank
*Vibrio toranzoniae* strain ENK1	*C. carcharias* cloaca	4,962,167	294,978	7.957	3	1,510,937	43.92	23.3	4,906	S RS21193063	JBLPGB000000000.1
*Aliivibrio fischeri ES114* strain ENK5	*C. carcharias* cloaca	4,750,424	735,427	8.892	11	2,972,306	38.42	52.4	4,982	SRS21193066	JBLOOV000000000.1
*Vibrio atlanticus* strain ENK4	*C. carcharias* skin	4,983,971	1,060,052	6.743	3	3,372,530	44.07	98.3	4,709	SRS21193065	CP171407.1 CP171408.1 CP171409.1
*Shewanella sp. WPAGA9* strain ENK2	*C. carcharias* skin	4,871,077	342,618	8.310	1	4,871,077	40.83	29	4,537	SRS21193064	CP171406.1
*Shewanella woodyi* strain ENK6	*C. brachyurus* cloaca	5,949,190	1,352,592	6.366	1	5,949,190	43.43	84	5,784	SRS21193067	CP171410.1

## Data Availability

Sequences are available under BioProject PRJNA1107358 (Table 1). Annotations are available on FigShare (https://figshare.com/articles/dataset/Bakta_annotations_for_bacteria_isolated_from_white_and_bronze_whaler_sharks/28702667).
